# Aspirin and other non-steroidal anti-inflammatory drugs and depression, anxiety, and stress-related disorders following a cancer diagnosis: a nationwide register-based cohort study

**DOI:** 10.1186/s12916-020-01709-4

**Published:** 2020-09-09

**Authors:** Kejia Hu, Arvid Sjölander, Donghao Lu, Adam K. Walker, Erica K. Sloan, Katja Fall, Unnur Valdimarsdóttir, Per Hall, Karin E. Smedby, Fang Fang

**Affiliations:** 1 Unit of Integrative Epidemiology, Institute of Environmental Medicine, Karolinska Institutet, Box 210, 171 77 Stockholm, Sweden; 2 Department of Medical Epidemiology and Biostatistics, Karolinska Institutet, Stockholm, Sweden; 3 Channing Division of Network Medicine, Brigham and Women’s Hospital, Harvard Medical School, Boston, MA USA; 4 Department of Epidemiology, Harvard T.H. Chan School of Public Health, Boston, MA USA; 5 Drug Discovery Biology Theme, Monash Institute of Pharmaceutical Sciences, Monash University, Parkville, Victoria 3052 Australia; 6 Laboratory of ImmunoPsychiatry, Neuroscience Research Australia, Randwick, New South Wales 2031 Australia; 7 School of Psychiatry, University of New South Wales, Sydney, 2052 Australia; 8 Clinical Epidemiology and Biostatistics School of Medical Sciences, Örebro Universitet, Örebro, Sweden; 9 Centre of Public Health Sciences Faculty of Medicine, University of Iceland, Reykjavík, Iceland; 10 Department of Oncology, Södersjukhuset, Stockholm, Sweden; 11 Division of Clinical Epidemiology, Department of Medicine Solna, Karolinska Institutet, Stockholm, Sweden

**Keywords:** Aspirin, Anti-inflammatory agents, non-steroidal, Mental disorders, Neoplasms

## Abstract

**Background:**

Cancer patients have a highly increased risk of psychiatric disorders following diagnosis, compared with cancer-free individuals. Inflammation is involved in the development of both cancer and psychiatric disorders. The role of non-steroidal anti-inflammatory drugs (NSAIDs) in the subsequent risk of psychiatric disorders after cancer diagnosis is however unknown.

**Methods:**

We performed a cohort study of all patients diagnosed with a first primary malignancy between July 2006 and December 2013 in Sweden. Cox proportional hazards models were used to assess the association of NSAID use during the year before cancer diagnosis with the risk of depression, anxiety, and stress-related disorders during the first year after cancer diagnosis.

**Results:**

Among 316,904 patients identified, 5613 patients received a diagnosis of depression, anxiety, or stress-related disorders during the year after cancer diagnosis. Compared with no use of NSAIDs, the use of aspirin alone was associated with a lower rate of depression, anxiety, and stress-related disorders (hazard ratio [HR], 0.88; 95% confidence interval [CI], 0.81 to 0.97), whereas the use of non-aspirin NSAIDs alone was associated with a higher rate (HR, 1.24; 95% CI, 1.15 to 1.32), after adjustment for sociodemographic factors, comorbidity, indications for NSAID use, and cancer characteristics. The association of aspirin with reduced rate of depression, anxiety, and stress-related disorders was strongest for current use (HR, 0.84; 95% CI, 0.75 to 0.93), low-dose use (HR, 0.88; 95% CI, 0.80 to 0.98), long-term use (HR, 0.84; 95% CI, 0.76 to 0.94), and among patients with cardiovascular disease (HR, 0.81; 95% CI, 0.68 to 0.95) or breast cancer (HR, 0.74; 95% CI, 0.56 to 0.98).

**Conclusion:**

Pre-diagnostic use of aspirin was associated with a decreased risk of depression, anxiety, and stress-related disorders during the first year following cancer diagnosis.

## Background

Psychiatric disorders are common comorbidities among patients with cancer [1] and may contribute to increased morbidity [2] and mortality [3, 4] after cancer diagnosis. In a previous study, we reported a highly increased risk of common psychiatric disorders, including depression, anxiety, and stress-related disorders, namely post-traumatic stress disorder (PTSD), acute stress reaction, adjustment disorder, and other stress reactions, among cancer patients, especially during the first year after cancer diagnosis [5]. Underlying reasons for such increased risk may include a severe stress response after receiving cancer diagnosis [6–8] and psychiatric symptoms caused by cancer treatment [9, 10], pain [11], and inflammation [12, 13]. Inflammation in the tumor micro-environment drives tumor development and progression [14] and cancer patients have a high burden of cancer-induced systemic inflammation [15].

Inflammation has been suggested to be involved in the development of depression [16] and other psychiatric disorders [17]. For instance, inflammatory cytokines have been shown to influence neurocircuitry in the brain through the consequence of neurotransmitter signaling [18], including a cascade of behavioral and immune responses that might lead to depression, anxiety [19], and PTSD [19, 20] among vulnerable individuals. Although the link between inflammation and psychiatric disorders has been less explored among patients with cancer, chronic inflammatory disorders have been reported to be risk factors for depression and anxiety among cancer patients [21].

Non-steroidal anti-inflammatory drugs (NSAIDs) are commonly prescribed for pain and inflammation. The anti-inflammatory actions of NSAIDs include inhibition of cyclooxygenase (COX) activity and prostaglandin synthesis [22]. Due to its non-competitive and irreversible acetylation of COX, aspirin is different from non-aspirin NSAIDs in terms of indications and adverse effects [23]. A recent meta-analysis of 26 relatively small randomized clinical trials suggested that NSAIDs play an antidepressant role in patients with major depressive disorder and are reasonably safe [24]. Preclinical studies indicate that the use of aspirin is associated with a lower risk of depression in the general population [25], and among patients with stroke [26] or osteoarthritis [27]. There is, however, a lack of evidence in this regard among cancer patients and on psychiatric disorders other than depression. Our recent preclinical study showed that low-dose aspirin might counteract the inflammation-related cognitive impairment in a mouse model of breast cancer [28]. It is therefore plausible that aspirin may help to prevent inflammation-related psychiatric disorders among cancer patients.

To this end, we performed a nationwide register-based study in Sweden to investigate the role of pre-diagnostic use of NSAIDs, especially aspirin, in the risk of depression, anxiety, and stress-related disorders following cancer diagnosis. Our hypothesis was that pre-diagnostic use of NSAIDs, especially aspirin, is associated with a decreased risk of depression, anxiety, and stress-related disorders following cancer diagnosis, compared with no use of NSAIDs.

## Methods

### Study design

**Fig. 1:**
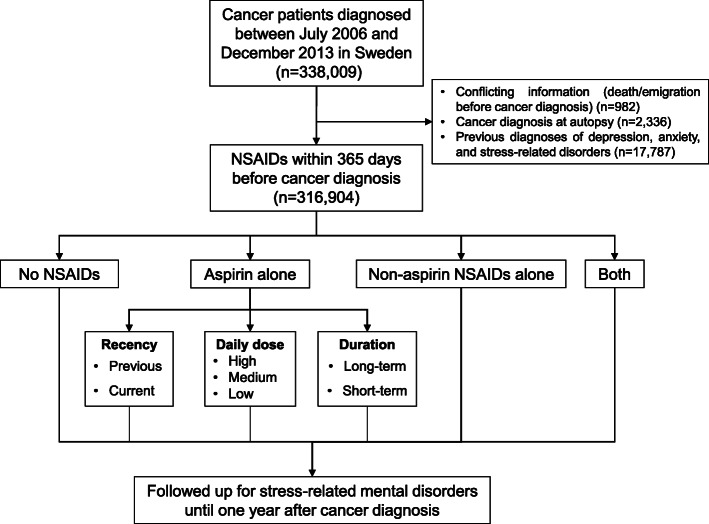
Flowchart of the study design. NSAIDs, non-steroidal anti-inflammatory drugs

From the Swedish Cancer Register [29], we identified 338,009 patients that were diagnosed with a first primary malignancy between July 1, 2006, and December 31, 2013. Through cross-linkages with the Swedish Causes of Death Register, Migration Register, and Patient Register, we followed these patients individually from the date of cancer diagnosis until death, emigration from Sweden, or 1 year after cancer diagnosis, whichever came first, using the Swedish personal identity numbers. We focused on the first year after cancer diagnosis because the risk of psychiatric disorders appears to be highest immediately after cancer diagnosis [5]. Patients were excluded from the analysis if they had conflicting information (i.e., died or emigrated before cancer diagnosis) (*n* = 982), or were diagnosed at autopsy (*n* = 2336). Because we aimed at assessing the risk of newly diagnosed psychiatric disorders, patients with preexisting depression, anxiety, or stress-related disorder before cancer diagnosis ascertained since 1973 onward according to the Patient Register [30] were also excluded (*n* = 17,787), leaving 316,904 patients in the final analysis (Fig. 1). We hypothesized the newly onset depression, anxiety, and stress-related disorders after cancer diagnosis might be more closely related to cancer-related inflammation and the psychological stress patients experienced after receiving a cancer diagnosis.

### Pre-diagnostic use of NSAIDs

We linked the cohort of cancer patients to the Swedish Prescribed Drug Register, which contains information on all prescribed medications that are dispensed in Sweden since July 2005 [31]. The vast majority of prescribed medications are subsidized with a celling of co-payment in Sweden. Medications are coded according to the Anatomical Therapeutic Chemical (ATC) Classification System in this register. We identified all records of NSAIDs (aspirin: N02BA01, N02BA51, B01AC06; non-aspirin NSAIDs: M01A) dispensed within 365 days before cancer diagnosis. Patients were then classified into the mutually exclusive categories “no use of NSAIDs,” “use of aspirin alone,” “use of non-aspirin NSAIDs alone,” or “use of both.” We also grouped the medications by cyclooxygenase selectivity. Aspirin, flurbiprofen, ketoprofen, fenoprofen, tolmetin, and oxaprozin were defined as COX-1 selective NSAIDs, whereas coxibs, meloxicam, etodolac, mefenamic acid, and diclofenac were defined as COX-2 selective NSAIDs. Nonselective NSAIDs and use of both categories were collapsed into an additional group.

As aspirin is the most frequently used NSAID, and our animal study suggested its protective role in cognitive well-being in a mouse model of breast cancer [28], we specifically studied aspirin and categorized the use of aspirin according to recency of use, daily dose, and duration of use. Recency of use was defined by the time between cancer diagnosis and last dispensation during 365 days before cancer diagnosis, and classified as “previous use” if the last dispensation was more than 90 days before cancer diagnosis or “current use” if no more than 90 days. The information of daily dose was extracted from the prescription text, and a mean daily dose was calculated from the prescribed daily dose of each dispensation. The daily dose was then categorized as low (20–150 mg), medium (151–300 mg), or high (> 300 mg). Duration of use was defined as the number of days that patients were supplied with aspirin, as estimated by the total dispensed dose divided by the mean daily dose. We defined a patient as a long-term user if the estimated duration was 300 days or more (the median of all duration values). Possible regimens of aspirin use, combining information on recency, dose, and duration, were also examined.

### Post-diagnostic depression, anxiety, and stress-related disorders

Through the Patient Register, we identified all patients with an inpatient or outpatient hospital visit that resulted in a diagnosis of psychiatric disorders [International Statistical Classification of Diseases and Related Health Problems 10th Revision (ICD-10): F10-F99] during the follow-up (from date of cancer diagnosis until up to 1 year after cancer diagnosis). We then limited the analysis to three groups of psychiatric disorders that are common among cancer patients [32, 33] and potentially related to inflammation [34], including (1) depression disorders (ICD-10: F32, F33), (2) anxiety disorders (ICD-10: F40, F41), and (3) stress-related disorders, namely PTSD, acute stress reaction, adjustment disorder, and other stress reactions (ICD-10: F43).

### Covariables

Information on age at diagnosis, date of diagnosis, cancer type, and cancer stage was obtained from the Cancer Register. We studied the most common cancer types, including prostate cancer, breast cancer, gastrointestinal cancers, lung cancer, skin cancer, kidney and bladder cancers, gynecological cancers, hematological malignancies, and other less common cancers including tumor of the central nervous system. Using the European Network of Cancer Registries Condensed TNM Scheme and International Federation of Gynecology and Obstetrics (FIGO) staging system, we classified non-hematological malignancies as localized limited (T-localized/N0/M0 or FIGO 0-I), localized advanced (T-advanced/N0/M0 or FIGO II), regional spread (any T/N+/M0 or FIGO III), distant metastasis (any T/any N/M+ or FIGO IV), and unknown stage. “Mx” was regarded as “M0” because “Mx” is commonly used when there are no clinical indications of distant metastasis.

We calculated chronic disease score based on the medications used during the year before cancer diagnosis, as a comorbidity measure [35, 36]. We excluded psychiatric medications, anti-inflammatory drugs, and analgesics from this calculation because they are related to the outcome and exposure of interest in the present study. We also ascertained potential indications for NSAID use through the Patient Register and classified them as cardiovascular diseases (ICD-10: G45.9, I00–02, I05–09, I20–21, I30, I32–33, I38–40, I63, I65.2, I80–83, I88, Z95), inflammatory musculoskeletal conditions (ICD-10: M05–19, M45–46, M60, M65), inflammatory systemic diseases (ICD-10: M30–36), and pain and fever (ICD-10: F45.4, G43–44, G50.0–50.1, M25.5, M54.5, M54.9, M79.1, N94.4, R50.8–50.9, R51–52). We further linked the cohort to the Longitudinal Integrated Database for Health Insurance and Labour Market Studies (LISA) [37] for ascertainment of various potential confounders, including educational level, occupation, region of residence (east, south, and north according to the first-level Classification of Territorial Units for Statistics), and marital status at cancer diagnosis.

### Statistical analysis

We described baseline characteristics of the cancer patients according to their levels of NSAID use. We calculated the incidence rates (IRs) of depression, anxiety, and stress-related disorders by diving the number of patients that received a diagnosis of such disorders by the accumulated number of person-years during follow-up. We then investigated the rate of the studied disorders in relation to the different levels of NSAID use using Cox proportional hazards models. In the Cox models, the “time to event” data were used by setting the date of cancer diagnosis (from the Cancer Register) as the time when an individual started being at risk for the outcome, the date of diagnosis of depression, anxiety, or stress-related disorder (from Patient Register) as the time of event, and the earliest date among death (from the Causes of Death Register), emigration (from the Migration Register), and 1 year after cancer diagnosis as the time of censoring if no event happened. We adjusted for age at cancer diagnosis, sex, calendar year of cancer diagnosis, educational level, occupation, place of residence, marital status, and comorbidity in model 1 and additionally adjusted for indications for NSAID use in model 2. We created one dummy variable per indication of NSAID use. In model 3, we further adjusted for cancer type and cancer stage. We first analyzed all studied disorders together and then analyzed separately depression, anxiety, and stress-related disorders. We then focused on aspirin and studied the effect of recency of use, daily dose, duration of use, and combined regimens of aspirin use. To calculate stratum-specific HRs, we fitted separate models for each level of sociodemographic factors, comorbidity, indications for use, and cancer characteristics for aspirin use as well as for current, low-dose, and long-term aspirin use. To test for effect modification by these variables, we included interaction terms between NSAID use and these variables in the multivariable models and used Wald tests to test the statistical significance of the estimated interaction terms (for one exposure and one stratification variable at a time).

Because patients with other psychiatric disorders might be more likely to use NSAIDs and at higher risk of studied disorders after cancer diagnosis, we performed a sensitivity analysis where cancer patients with any pre-existing psychiatric disorders before cancer diagnosis were excluded. Additionally, as patients with gastrointestinal symptoms might avoid the use of NSAIDs and might have a higher risk of depression, anxiety, and stress-related disorders in general, we conducted another sensitivity analysis where we stratified the analysis by use of proton pump inhibitors, defined through at least two dispensations, within 1 year before cancer diagnosis. In all analyses, time since cancer diagnosis was used as the underlying timescale. We used Schoenfeld residuals to test the proportional hazards assumption for the main exposure (i.e., NSAID use) in all models and found no major deviation from the assumption.

The statistical analyses were performed using SAS, version 9.4, SAS Institute and Stata, version 16, StataCorp LP. We used a two-sided *P* < 0.05 to indicate statistical significance.

## Results

Among the 316,904 patients included in the analysis, the median age at cancer diagnosis was 68 years and 53.4% were male. 58,761 (18.5%) of these patients used aspirin alone and 49,059 (15.4%) used non-aspirin NSAIDs alone during the year before cancer diagnosis (Fig. 1). Cancer patients who used aspirin alone were more likely to be male, older, less educated, were less likely to be working, had more comorbidities, and were more likely to have a deceased partner, compared with patients who did not use any NSAIDs (Table 1). Compared with no users, aspirin users were more likely to have cardiovascular disease, whereas non-aspirin NSAID users were more likely to have inflammatory musculoskeletal conditions such as arthritis.

**Table 1:** Baseline characteristics of cancer patients at the time of cancer diagnosis by exclusive use of NSAIDs

Characteristics	No NSAIDs	Aspirin	Non-aspirin NSAIDs	Both NSAIDs
Number	194,198	58,761	49,059	14,886
Year of cancer diagnosis (%)				
2006–2009	86,249 (44.4)	26,731 (45.5)	23,287 (47.5)	7411 (49.8)
2010–2013	107,949 (55.6)	32,030 (54.5)	25,772 (52.5)	7475 (50.2)
Sex (%)				
Male	99,678 (51.3)	36,861 (62.7)	23,780 (48.5)	8910 (59.9)
Female	94,520 (48.7)	21,900 (37.3)	25,279 (51.5)	5976 (40.1)
Age at cancer diagnosis, mean [SD], years (%)	65.7 [13.0]	75.4 [9.5]	64.8 [11.8]	73.3 [9.2]
Educational level^a^ (%)				
Low	63,369 (32.6)	27,762 (47.2)	15,859 (32.3)	6546 (44.0)
Medium	76,981 (39.6)	20,699 (35.2)	20,420 (41.6)	5590 (37.6)
High	51,399 (26.5)	9267 (15.8)	12,221 (24.9)	2513 (16.9)
Unknown	2449 (1.3)	1033 (1.8)	559 (1.1)	237 (1.6)
Occupation (%)				
Blue-collar	28,227 (14.5)	2772 (4.7)	8472 (17.3)	901 (6.1)
White-collar	46,049 (23.7)	4163 (7.1)	10,959 (22.3)	1215 (8.2)
Not working	119,190 (61.4)	51,743 (88.1)	29,460 (60.1)	12,742 (85.6)
Unclassified or unknown	732 (0.4)	83 (0.1)	168 (0.3)	28 (0.2)
Region of residence^b^ (%)				
East	71,206 (36.7)	19,957 (34.0)	17,831 (36.3)	4903 (32.9)
South	87,891 (45.3)	26,708 (45.5)	22,503 (45.9)	6834 (45.9)
North	35,101 (18.1)	12,096 (20.6)	8725 (17.8)	3149 (21.2)
Marital status (%)				
Unmarried	29,383 (15.1)	4990 (8.5)	6355 (13.0)	1101 (7.4)
Married/registered partnership	108,123 (55.7)	31,120 (53.0)	28,141 (57.4)	8242 (55.4)
Divorced/separated	30,803 (15.9)	8474 (14.4)	8734 (17.8)	2441 (16.4)
Widow(er)/surviving partner	25,889 (13.3)	14,177 (24.1)	5829 (11.9)	3102 (20.8)
Chronic disease score^c^ (%)				
0	80,567 (41.5)	25 (0.0)	15,061 (30.7)	19 (0.1)
1–3	95,079 (49.0)	25,849 (44.0)	29,061 (59.2)	5792 (38.9)
> 3	18,552 (9.6)	32,887 (56.0)	4937 (10.1)	9075 (61.0)
Diagnosis of potential indications for NSAIDs^d^ (%)				
Cardiovascular disease	30,121 (15.5)	28,326 (48.2)	7573 (15.4)	6823 (45.8)
Inflammatory musculoskeletal condition	28,002 (14.4)	10,981 (18.7)	14,905 (30.4)	4984 (33.5)
Inflammatory systemic disease	2792 (1.4)	1296 (2.2)	1135 (2.3)	462 (3.1)
Pain and fever	2410 (1.2)	630 (1.1)	1044 (2.1)	259 (1.7)
Cancer stage^e^ (%)				
Localized limited	57,011 (29.4)	14,106 (24.0)	13,274 (27.1)	3474 (23.3)
Localized advanced	11,719 (6.0)	3906 (6.6)	2328 (4.7)	743 (5.0)
Regional spread	20,914 (10.8)	5571 (9.5)	4730 (9.6)	1314 (8.8)
Distant metastasis	19,155 (9.9)	6317 (10.8)	5954 (12.1)	1934 (13.0)
Unknown	72,027 (37.1)	23,868 (40.6)	18,232 (37.2)	5929 (39.8)
Not applicable^f^	13,372 (6.9)	4993 (8.5)	4541 (9.3)	1492 (10.0)
Cancer type^g^ (%)				
Prostate cancer	37,380 (19.2)	12,510 (21.3)	9699 (19.8)	3309 (22.2)
Breast cancer	30,135 (15.5)	4702 (8.0)	7484 (15.3)	1313 (8.8)
Gastrointestinal cancer	34,625 (17.8)	11,340 (19.3)	7029 (14.3)	2381 (16.0)
Lung cancer	11,126 (5.7)	4612 (7.8)	3867 (7.9)	1426 (9.6)
Non-melanoma skin cancer	11,619 (6.0)	5612 (9.6)	2190 (4.5)	1057 (7.1)
Melanoma	11,417 (5.9)	2242 (3.8)	2233 (4.6)	547 (3.7)
Kidney & bladder cancer	10,785 (5.6)	4764 (8.1)	2735 (5.6)	1155 (7.8)
Gynecological cancer	11,313 (5.8)	2438 (4.1)	2901 (5.9)	615 (4.1)
Hematological malignancy	13,372 (6.9)	4993 (8.5)	4541 (9.3)	1492 (10.0)
Other cancers	22,426 (11.5)	5548 (9.4)	6380 (13.0)	1591 (10.7)

Cancer patients were categorized into exclusive groups according to their usage of NSAIDs during the year before cancer diagnosis: non-users (never used any NSAID), aspirin users (used aspirin but not non-aspirin), non-aspirin NSAID users (used non-aspirin NSAIDs but not aspirin), users of both NSAIDs (used both aspirin and non-aspirin NSAIDs)

^a^Classified according to years of education: high (college and above), medium (9 years plus 2–3 years secondary school), low (9 years or less), or unknown

^b^Identified through the First-level Classification of Territorial Units for Statistics, NUTS-1

^c^Calculated based on all medications used within 1 year before cancer diagnosis, after excluding psychiatric medications, anti-inflammatory drugs, and analgesics from the original codes list that are directly related to the outcome or exposure of interest

^d^Identified from the Swedish Patient Register since 2001. The four groups of potential indications are non-exclusive

^e^Defined by European Network of Cancer Registries Condensed TNM Scheme and International Federation of Gynecology and Obstetrics staging system: localized limited (T-localized/N0/M0 or FIGO 0-I), localized advanced (T-advanced/N0/M0 or FIGO II), regional spread (any T/N+/M0 or FIGO III), distant metastasis (any T/any N/M+ or FIGO IV), or unknown stage

^f^Hematological malignancies were further divided into five subtypes: leukemia, lymphoma, myeloma, myelodysplastic syndrome, and myeloproliferative neoplasm

^g^Displayed according to the most common cancer types in this population

### Types of NSAID use and risk of depression, anxiety, and stress-related disorders

A total of 5613 patients received a diagnosis of depression, anxiety, and stress-related disorders during the year after cancer diagnosis. Compared with no NSAID use, the use of aspirin alone was associated with a lower rate of the studied disorders in all models (HR, 0.88; 95% CI, 0.81 to 0.97 in model 3 with adjustment for sociodemographic factors, comorbidity, indications, and cancer characteristics) (Table 2). Similar results were found for depression, anxiety, and stress-related disorders separately (Supplementary Table S1). In contrast, the use of non-aspirin NSAIDs alone was associated with an increased rate of the studied disorders (Table 2 and Supplementary Table S1), compared with no NSAID use. We did not find an association between the use of both aspirin and non-aspirin NSAIDs and the studied disorders. The sensitivity analyses showed similar results after excluding patients with any pre-existing psychiatric disorders (Supplementary Table S2) or after stratifying the analysis by use of proton pump inhibitors within 1 year before cancer diagnosis (Supplementary Table S3).

**Table 2:** Hazard ratios (95% confidence intervals) of depression, anxiety, and stress-related disorders during the year after cancer diagnosis, in relation to pre-diagnostic use of NSAIDs

Group	1000 PYs	Event (IR)	Model 1^a^	Model 2^b^	Model 3^c^
No NSAIDs	174	3408 (19.6)	1.00	1.00	1.00
Aspirin	50	797 (16.1)	0.88 (0.80–0.96)	0.86 (0.79–0.95)	0.88 (0.81–0.97)
Non-aspirin NSAIDs	43	1145 (26.4)	1.26 (1.18–1.35)	1.27 (1.18–1.36)	1.24 (1.15–1.32)
Both NSAIDs	13	263 (20.9)	1.05 (0.91–1.20)	1.04 (0.91–1.19)	1.05 (0.92–1.20)

Cancer patients with any diagnosis of depression, anxiety, or stress-related disorders, namely post-traumatic stress disorder, acute stress reaction, adjustment disorder, or other stress reactions before cancer diagnosis were excluded from the analysis. IRs were calculated by dividing the number of patients that received a diagnosis of depression, anxiety, and stress-related disorders by the number of person-years accumulated during follow-up. Multivariable Cox proportional hazard models were used to estimate the hazard ratios and 95% confidence intervals in relation to exclusive NSAID use, using no use of NSAIDs as the reference

*Abbreviations*: *PYs* person-years, *IR* incidence rate per 1000 person-years

^a^Estimates were adjusted for age, sex, calendar year of cancer diagnosis, educational level, occupation, region of residence, marital status, and Chronic Disease Score

^b^Estimates were additionally adjusted for potential indications for NSAID use

^c^Estimates were additionally adjusted for common cancer types and cancer stage, as well as subtypes of hematological malignancies (leukemia, lymphoma, myeloma, myelodysplastic syndrome, or myeloproliferative neoplasm)

A lower rate of depression, anxiety, and stress-related disorders, although not statistically significant, was suggested for use of COX-1 selective NSAIDs (HR, 0.93; 95% CI, 0.85–1.01) compared with no NSAID use (Supplementary Table S4). The use of COX-2 selective NSAIDs was associated with a higher rate (HR, 1.26; 95% CI, 1.15–1.38) of the studied disorders compared with no NSAID use. A combined use of COX-1 selective, COX-2 selective, and nonselective NSAIDs was associated with a moderately increased rate of the studied disorders.

### Recency, daily dose, and duration of aspirin use

The association of aspirin with reduced rate of depression, anxiety, and stress-related disorders was primarily noted among current users (HR, 0.84; 95% CI, 0.75 to 0.93), low-dose users (HR, 0.88; 95% CI, 0.80 to 0.98), and long-term users (HR, 0.84; 95% CI, 0.76 to 0.94) (Table 3). Among commonly combined regimens, individuals with a combination of current, low-dose, and long-term aspirin use had the lowest rate of the studied disorders after cancer diagnosis (HR, 0.77; 95% CI, 0.68 to 0.87).

**Table 3:** Hazard ratios (95% confidence intervals) of depression, anxiety, and stress-related disorders during the year after cancer diagnosis in relation to pre-diagnostic exclusive use of aspirin, analysis by recency of use, daily dose, and duration of use

Characteristics	1000 PYs	Event (IR)	Model 1^a^	Model 2^b^	Model 3^c^
**No NSAIDs (reference group)**	174	3408 (19.6)	1.00	1.00	1.00
**Recency of aspirin use**					
Previous	12	229 (19.1)	1.02 (0.89–1.18)	1.02 (0.88–1.18)	1.01 (0.88–1.17)
Current	38	568 (15.1)	0.82 (0.74–0.91)	0.81 (0.73–0.90)	0.84 (0.75–0.93)
**Daily dose of aspirin use**					
Low-dose	40	659 (16.4)	0.87 (0.79–0.97)	0.87 (0.78–0.96)	0.88 (0.80–0.98)
Medium dose	6	90 (15.8)	0.95 (0.76–1.18)	0.94 (0.75–1.17)	0.96 (0.77–1.19)
High dose	1	18 (19.9)	1.17 (0.73–1.86)	1.16 (0.72–1.84)	1.17 (0.73–1.86)
**Duration of aspirin use**					
Short-term	10	210 (20.6)	1.07 (0.93–1.25)	1.06 (0.92–1.24)	1.07 (0.92–1.25)
Long-term	37	557 (15.2)	0.83 (0.74–0.92)	0.82 (0.74–0.91)	0.84 (0.76–0.94)
**Combined regimens**					
Previous, low-dose	10	195 (19.3)	1.03 (0.88–1.21)	1.02 (0.87–1.19)	1.02 (0.87–1.19)
Previous, medium-high dose	2	30 (17.8)	1.09 (0.75–1.57)	1.06 (0.74–1.53)	1.04 (0.72–1.49)
Current, low-dose, long-term	26	362 (14.2)	0.76 (0.67–0.86)	0.75 (0.66–0.85)	0.77 (0.68–0.87)
Current, low-dose, short-term	5	102 (22.1)	1.15 (0.94–1.41)	1.13 (0.92–1.38)	1.16 (0.94–1.42)
Current, medium-high dose, long-term	4	65 (15.5)	0.94 (0.73–1.21)	0.92 (0.71–1.18)	0.95 (0.74–1.23)
Current, medium-high dose, short-term	1	13 (18.1)	1.04 (0.60–1.80)	1.02 (0.59–1.76)	1.05 (0.61–1.82)

Recency was defined by the time since last dispensed date to cancer diagnosis and patients were classified into previous users (more than 90 days before cancer diagnosis) and current users (within 90 days before cancer diagnosis). A total dispensed dose of aspirin, average daily dose, and number of days to be covered were calculated according to prescription text. Average daily dose was then categorized as low (20–150 mg), medium (151–300 mg), and high (> 300 mg) dose. Long-term users were defined as those who used aspirin for ≥ 300 days during the 365 days before cancer diagnosis. IRs were calculated by dividing the number of patients that received a diagnosis of depression, anxiety, and stress-related disorders by the number of person-years accumulated during follow-up. Combined regimens across recency, dose, and duration were also examined. Hazard ratios and 95% confidence intervals were estimated from separate Cox proportional hazard models to assess the effect of recency, dose, and duration of use, as well as the six combined regimens of aspirin use, using no use of NSAIDs as the reference

*Abbreviations*: *PYs* person-years, *IR* incidence rate per 1000 person-years

^a^Estimates were adjusted for age, sex, calendar year at cancer diagnosis, educational level, occupation, region of residence, marital status, and Chronic Disease Score

^b^Estimates were additionally adjusted for potential indications for NSAID use

^c^Estimates were additionally adjusted for common cancer types and cancer stage, as well as subtypes of hematological malignancies (leukemia, lymphoma, myeloma, myelodysplastic syndrome, or myeloproliferative neoplasm)

### Subgroup analysis and effect modification

The magnitude of the inverse association of aspirin use alone with the rate of depression, anxiety, and stress-related disorders was greater among females than males, among patients who lived in the east of Sweden than patients who lived in other parts of Sweden, among patients with cardiovascular disease than patients without cardiovascular disease, and among breast cancer patients than patients with other cancer types (all *P* values for interaction < 0.05) (Table 4). The association did not, however, differ by calendar period of cancer diagnosis, age, educational level, marital status, occupation, comorbidity, other indications for NSAID use, or cancer stage. Similar result patterns were found for current use, long-term use, and low-dose use of aspirin.

**Table 4:** Hazard ratios (95% confidence intervals) of depression, anxiety, and stress-related disorders during the year after cancer diagnosis in relation to pre-diagnostic exclusive use of aspirin, stratified analysis by different factors

Characteristics	Any exclusive aspirin use	Current aspirin	Low-dose aspirin	Long-term aspirin
**Calendar year at cancer diagnosis**				
2006–2009	0.82 (0.71–0.95)	0.74 (0.63–0.87)	0.82 (0.70–0.97)	0.77 (0.65–0.92)
2010–2013	0.93 (0.82–1.06)	0.90 (0.78–1.04)	0.93 (0.81–1.06)	0.89 (0.77–1.03)
*P* for interaction	0.72	0.36	0.66	0.70
**Sex**				
Male	0.96 (0.83–1.11)	0.92 (0.79–1.07)	0.97 (0.83–1.12)	0.90 (0.77–1.06)
Female	0.80 (0.70–0.92)	0.74 (0.63–0.86)	0.80 (0.69–0.93)	0.77 (0.65–0.90)
*P* for interaction	< 0.01	< 0.01	< 0.01	0.01
**Age at cancer diagnosis, years**				
< 50	0.80 (0.42–1.54)	0.80 (0.38–1.71)	0.86 (0.44–1.71)	0.87 (0.37–2.07)
50–59	0.89 (0.65–1.21)	0.79 (0.55–1.13)	0.87 (0.63–1.20)	0.71 (0.48–1.05)
60–69	0.89 (0.74–1.08)	0.88 (0.71–1.08)	0.91 (0.75–1.11)	0.84 (0.68–1.04)
≥ 70	0.90 (0.79–1.03)	0.84 (0.73–0.97)	0.91 (0.79–1.04)	0.89 (0.77–1.02)
*P* for interaction	0.82	0.92	0.93	0.32
**Education level**				
Low	0.89 (0.76–1.03)	0.85 (0.72–1.01)	0.89 (0.76–1.05)	0.87 (0.74–1.03)
Medium	1.02 (0.87–1.20)	0.93 (0.78–1.12)	1.04 (0.88–1.23)	0.96 (0.80–1.15)
High	0.67 (0.53–0.84)	0.66 (0.51–0.85)	0.63 (0.49–0.81)	0.58 (0.45–0.77)
*P* for interaction	0.14	0.58	0.05	0.07
**Marital status**				
Unmarried	0.72 (0.54–0.96)	0.76 (0.55–1.04)	0.78 (0.58–1.06)	0.72 (0.52–1.01)
Married/registered partnership	0.86 (0.74–0.99)	0.79 (0.67–0.93)	0.84 (0.72–0.98)	0.78 (0.66–0.91)
Divorced/separated	0.97 (0.78–1.20)	0.96 (0.75–1.22)	0.98 (0.78–1.23)	0.99 (0.77–1.26)
Widow(er)/surviving partner	0.97 (0.78–1.20)	0.88 (0.69–1.11)	0.98 (0.78–1.23)	0.95 (0.75–1.20)
*P* for interaction	0.82	0.78	0.76	0.27
**Occupation**				
Blue-collar	0.89 (0.61–1.29)	0.80 (0.52–1.23)	0.88 (0.60–1.30)	0.86 (0.56–1.32)
White-collar	0.80 (0.58–1.11)	0.74 (0.51–1.07)	0.77 (0.55–1.09)	0.60 (0.40–0.91)
Not working	0.89 (0.80–1.00)	0.85 (0.75–0.95)	0.90 (0.81–1.01)	0.87 (0.77–0.97)
*P* for interaction	0.93	0.92	0.86	0.84
**Place of residence**				
East	0.83 (0.71–0.97)	0.76 (0.64–0.91)	0.86 (0.73–1.01)	0.79 (0.67–0.94)
South	0.93 (0.81–1.08)	0.93 (0.79–1.10)	0.93 (0.80–1.09)	0.90 (0.76–1.06)
North	0.87 (0.69–1.12)	0.73 (0.56–0.97)	0.84 (0.65–1.09)	0.80 (0.61–1.05)
*P* for interaction	< 0.01	< 0.01	0.01	0.01
**Chronic disease score**^***c***^				
1–3	0.91 (0.79–1.04)	0.84 (0.72–0.98)	0.91 (0.79–1.05)	0.86 (0.74–1.01)
> 3	0.87 (0.75–1.00)	0.83 (0.71–0.97)	0.88 (0.75–1.02)	0.82 (0.70–0.96)
*P* for interaction	0.39	0.59	0.38	0.35
**Cardiovascular disease**				
No	0.95 (0.84–1.07)	0.88 (0.76–1.01)	0.97 (0.85–1.10)	0.90 (0.78–1.03)
Yes	0.81 (0.68–0.95)	0.79 (0.66–0.94)	0.79 (0.66–0.93)	0.78 (0.66–0.93)
*P* for interaction	0.01	0.12	0.01	0.07
**Inflammatory musculoskeletal condition**				
No	0.90 (0.81–1.01)	0.84 (0.74–0.95)	0.92 (0.82–1.03)	0.86 (0.76–0.97)
Yes	0.82 (0.66–1.02)	0.81 (0.64–1.03)	0.76 (0.60–0.96)	0.78 (0.61–0.99)
*P* for interaction	0.49	0.90	0.16	0.54
**Inflammatory systemic disease**				
No	0.88 (0.80–0.97)	0.83 (0.74–0.93)	0.89 (0.80–0.98)	0.84 (0.75–0.94)
Yes	0.93 (0.51–1.69)	0.85 (0.43–1.67)	0.80 (0.41–1.57)	0.75 (0.37–1.54)
*P* for interaction	0.88	0.85	0.47	0.58
**Pain and fever**				
No	0.88 (0.80–0.97)	0.83 (0.74–0.92)	0.88 (0.79–0.98)	0.84 (0.75–0.94)
Yes	0.98 (0.52–1.84)	1.04 (0.52–2.06)	0.92 (0.47–1.80)	0.61 (0.28–1.36)
*P* for interaction	0.90	0.66	0.98	0.42
**Cancer stage**				
Localized limited	0.78 (0.63–0.95)	0.71 (0.56–0.89)	0.79 (0.64–0.97)	0.73 (0.58–0.92)
Localized advanced	1.06 (0.75–1.50)	1.12 (0.77–1.64)	0.95 (0.65–1.39)	0.82 (0.55–1.24)
Regional spread	0.78 (0.61–0.99)	0.72 (0.55–0.95)	0.77 (0.60–1.00)	0.73 (0.56–0.96)
Distant metastasis	1.01 (0.76–1.34)	0.98 (0.72–1.33)	1.10 (0.83–1.47)	1.08 (0.80–1.47)
*P* for interaction	0.57	0.45	0.23	0.14
**Cancer type**				
Prostate cancer	0.94 (0.71–1.26)	0.88 (0.64–1.20)	0.93 (0.68–1.26)	0.77 (0.56–1.07)
Breast cancer	0.74 (0.56–0.98)	0.63 (0.46–0.87)	0.73 (0.54–0.97)	0.71 (0.52–0.98)
Gastrointestinal cancer	1.03 (0.84–1.27)	1.05 (0.84–1.32)	1.02 (0.82–1.27)	1.03 (0.82–1.29)
Lung cancer	0.78 (0.58–1.05)	0.70 (0.50–0.97)	0.82 (0.61–1.12)	0.73 (0.53–1.01)
Skin cancer excl. Melanoma	1.07 (0.66–1.74)	0.89 (0.52–1.53)	1.09 (0.65–1.84)	1.19 (0.71–1.99)
Melanoma	0.56 (0.30–1.03)	0.54 (0.28–1.05)	0.45 (0.23–0.91)	0.42 (0.20–0.89)
Kidney and bladder	0.86 (0.59–1.26)	0.80 (0.52–1.23)	0.85 (0.56–1.27)	0.92 (0.61–1.39)
Gynecologic cancer	0.76 (0.52–1.12)	0.83 (0.55–1.25)	0.78 (0.52–1.16)	0.68 (0.44–1.05)
Hematological malignancies	1.16 (0.82–1.66)	1.25 (0.85–1.83)	1.25 (0.87–1.80)	1.18 (0.79–1.76)
Other cancers	0.80 (0.62–1.05)	0.67 (0.49–0.91)	0.83 (0.63–1.09)	0.74 (0.55–1.01)
*P* for interaction	0.04	0.03	0.15	0.12

The columns refer to four definitions of exposure in separate models: any exclusive use of aspirin, current use of aspirin, low-dose use of aspirin, and long-term use of aspirin, where no use of NSAIDs was used as the reference in all models. Stratum-specific hazard ratios were estimated for each exposure by fitting separate Cox proportional hazard models for different levels of the stratification variable and are presented in different rows, adjusting for age, sex, calendar year at cancer diagnosis, educational level, occupation, region of residence, marital status, Chronic Disease Score, potential indications for NSAIDs, cancer type, cancer stage, and subtypes of hematological malignancies. To assess the interaction between the exposure and each stratification variable, multivariable models were fitted adjusting for the above covariates and including an interaction term for the exposure and each level of the individual stratification variable. P for interaction was then calculated through a Wald test of the null hypothesis that interaction parameter for the exposure and the individual stratification variable is equal to zero. *P* < 0.05 indicates a statistically significant interaction between the exposure and the stratification variable

## Discussion

To the best of our knowledge, this nationwide register-based cohort study is the first to examine the association of prior NSAID use with the risk of depression, anxiety, and stress-related disorders after cancer diagnosis. The study included all patients with newly diagnosed cancer in Sweden between July 2006 and December 2013 and found that aspirin use, especially long-term and low-dose use, shortly before cancer diagnosis was associated with a reduced rate of depression, anxiety, and stress-related disorders during the first year after cancer diagnosis. Non-aspirin NSAID use, on the other hand, was associated with a higher rate of depression, anxiety, and stress-related disorders.

The different results for aspirin and non-aspirin NSAIDs are consistent with recent studies of depression in the general population [25] and among patients with stroke [26]. We further extended the knowledge to other common psychiatric disorders, including anxiety and stress-related disorders (namely PTSD, acute stress reaction, adjustment disorder, and other stress reactions). The different results for aspirin and non-aspirin NSAIDs are also biologically plausible. Accumulating evidence supports the role of COX-1 inhibition in attenuating neuroinflammation, leading to protection against inflammatory brain damage [28, 38, 39]. In contrast, COX-2 inhibitors have been shown to augment nitro-oxidative and oxidative stress in the brain [40], and to interfere with the resolution of inflammation by decreasing the negative immunoregulator Prostaglandin E2 [41], thereby aggravate neuroinflammation. Consistent with this, selective COX-2 inhibitors have also been related to increased psychiatric symptoms including depression, anxiety, and changes in cognition [42]. Taken together, COX-1, rather than COX-2 inhibition, might be the key factor in blocking neuroinflammation [38]. Our findings that COX-2 selective NSAID use was associated with a higher risk of common psychiatric disorders compared with nonselective NSAID use also supports this hypothesis. Aspirin selectively inhibits COX-1, particularly at a low dose [38]. As the majority of aspirin is used at low dose, the observed protective effect of aspirin on the studied disorders is likely driven by COX-1 inhibition. The null association of high-dose aspirin may on the other hand be due to additional inhibition of COX-2 [25]. In contrast, although non-aspirin NSAIDs have mixed selectivity, they are mostly selective for COX-2 inhibition or non-selective, possibly leading to the observed harmful effect of non-aspirin NSAIDs on these psychiatric disorders. This was however partly inconsistent with the findings of a recent meta-analysis that found celecoxib to have an antidepressant effect when added to traditional antidepressants [24]. One possible explanation for the contradictory findings might be the fact that we studied newly onset depression, anxiety, and stress-related disorders after cancer diagnosis whereas Bai et al. studied the treatment effort on prevalent depression [24].

We further found that current, long-term, and low-dose aspirin use was associated with the lowest rate of depression, anxiety, and stress-related disorders after cancer diagnosis. Current and long-term aspirin use reflects persistent anti-inflammatory activity, in addition to low-dose use with enhanced inhibition of COX-1. The stratified results by cancer stage showed clear evidence for an inverse association between aspirin and the studied disorders in most stages of cancer, apart from distant metastatic cancer. Furthermore, the effect of aspirin was more pronounced among females, in line with the proposed female-specific impact of low-dose aspirin in anti-inflammation [43]. The stronger association noted for females might alternatively be attributable to the stronger findings among breast cancer patients, which corroborates with our preclinical findings using animal models [28]. The underlying reasons for the stronger association among patients with breast cancer, compared with patients with other cancers, remain unknown. This finding, together with the fact that patients with breast cancer have indeed the highest prevalence of depression among patients of different cancer types [33], highlights a potentially specific role of inflammation in breast cancer-related depression [44].

The strength of the study includes the nationwide population-based study design, independent collection of information on drug exposures and psychiatric disorders, the comprehensive information of covariables, and thorough statistical analysis including multivariable adjustments for potential confounders. Common systemic and random errors are therefore minimized. Some potential limitations of the study should, however, still be noted. The Prescribed Drug Register does not include information on medications used over the counter or in hospitals and nursing homes. We speculate however that this is non-differential between individuals that would later receive a cancer diagnosis and subsequently also a diagnosis of depression, anxiety, and stress-related disorders and individuals that would not receive a diagnosis of psychiatric disorders after being diagnosed with cancer. Such misclassification would therefore most likely have diluted the magnitude of the studied association. Also, this should not have affected the results on aspirin use greatly because low-dose aspirin is mostly prescribed in Sweden [45]. Further, because of its observational nature, residual confounding due to unknown and unmeasured confounders may exist. Patients with a pre-existing psychiatric disorder might be more likely to use NSAIDs and at higher risk of depression, anxiety, and stress-related disorders after cancer diagnosis. We therefore excluded patients with any psychiatric disorders—in addition to patients with depression, anxiety, and stress-related disorders—prior to cancer diagnosis and observed similar results. However, the exclusion of these patients was likely incomplete because not all patients with psychiatric disorders attend health care. Similarly, we investigated the role of gastrointestinal symptoms, which could indicate high stress levels and reduced NSAID use, by stratifying the analysis by use of proton pump inhibitors during the year before cancer diagnosis and found again similar results.

## Conclusions

Aspirin use, especially current, long-term, and low-dose use, was associated with a decreased risk of depression, anxiety, and stress-related disorders following cancer diagnosis, while the use of non-aspirin NSAIDs was associated with an increased risk, compared with no use of NSAIDs. Our findings call for pre-clinical research in examining the underlying mechanisms of low-dose aspirin and depression, anxiety, and stress-related disorders after cancer diagnosis and, if confirmed in further studies, provide a rationale for randomized clinical trials.

## Supplementary information

**Additional file 1: Table S1.** Hazard ratios (95% confidence intervals) of depression, anxiety, or stress-related disorder during the year after cancer diagnosis in relation to pre-diagnostic use of NSAIDs. **Table S2.** Hazard ratios (95% confidence intervals) of depression, anxiety, and stress-related disorders during the year after cancer diagnosis in relation to pre-diagnostic use of NSAIDs, after excluding patients with any psychiatric disorders before cancer diagnosis. **Table S3.** Hazard ratios (95% confidence intervals) of depression, anxiety, and stress-related disorders during the year after cancer diagnosis in relation to pre-diagnostic NSAID use, stratified analysis by pre-diagnostic use of proton pump inhibitors (PPIs). **Table S4.** Hazard ratios (95% confidence intervals) of depression, anxiety, and stress-related disorders during the year after cancer diagnosis in relation to pre-diagnostic use of NSAIDs, analysis by selectivity of NSAIDs.

## Data Availability

Data are from the Swedish Cancer Register, Causes of Death Register, Migration Register, Patient Register, and Prescribed Drug Register. Data cannot be put into a public data repository due to Swedish law but are available by applying through Statistics Sweden (for Migration Register) and the Swedish National Board of Health and Welfare (for the other four registers). Detailed information on data application is in the following links: https://www.scb.se/vara-tjanster/bestalla-mikrodata/ and https://bestalladata.socialstyrelsen.se/.

## Abbreviations

CI: Confidence interval
COX: Cyclooxygenase
FIGO: International Federation of Gynecology and Obstetrics
HR: Hazard ratio
ICD-10: International Statistical Classification of Diseases and Related Health Problems 10th Revision
LISA: Longitudinal Integrated Database for Health Insurance and Labour Market Studies
NSAIDs: Non-steroidal anti-inflammatory drugs
PTSD: Post-traumatic stress disorder
