# Supplementary material for: Aspirin and other non-steroidal anti-inflammatory drugs and depression, anxiety, and stress-related disorders following a cancer diagnosis: a nationwide register-based cohort study
Source: BMC Med. 2020 Sep 9;18:238. doi: 10.1186/s12916-020-01709-4 (PMC7487710; doi:10.1186/s12916-020-01709-4)
Supplement: Supplementary file 1 — Additional file 1: Table S1. Hazard ratios (95% confidence intervals) of depression, anxiety, or stress-related disorder during the year after cancer diagnosis in relation to pre-diagnostic use of NSAIDs. Table S2. Hazard ratios (95% confidence intervals) of depression, anxiety, and stress-related disorders during the year after cancer diagnosis in relation to pre-diagnostic use of NSAIDs, after excluding patients with any psychiatric disorders before cancer diagnosis. Table S3. Hazard ratios (95% confidence intervals) of depression, anxiety, and stress-related disorders during the year after cancer diagnosis in relation to pre-diagnostic NSAID use, stratified analysis by pre-diagnostic use of proton pump inhibitors (PPIs). Table S4. Hazard ratios (95% confidence intervals) of depression, anxiety, and stress-related disorders during the year after cancer diagnosis in relation to pre-diagnostic use of NSAIDs, analysis by selectivity of NSAIDs. [file 12916_2020_1709_MOESM1_ESM.docx]

Aspirin and other non-steroidal anti-inflammatory drugs and depression, anxiety, and stress-related disorders following a cancer diagnosis: a nationwide register-based cohort study

Kejia Hu^1^, Arvid Sjölander^2^, Donghao Lu^2,3,4^, Adam K. Walker^5,6,7^, Erica K. Sloan^5^, Katja Fall^1,8^, Unnur Valdimarsdóttir^2,9^, Per Hall^2,10^, Karin E. Smedby^11^, Fang Fang^1,^*

**Supplementary Table S1.** **Hazard ratios (95% confidence intervals) of depression, anxiety, or stress-related disorder during the year after cancer diagnosis in relation to pre-diagnostic use of NSAIDs**

**Supplementary Table S2. Hazard ratios (95% confidence intervals) of depression, anxiety, and stress-related disorders during the year after cancer diagnosis in relation to pre-diagnostic use of NSAIDs, after excluding patients with any psychiatric disorders before cancer diagnosis**

**Supplementary Table S3. Hazard ratios (95% confidence intervals) of depression, anxiety, and stress-related disorders during the year after cancer diagnosis in relation to pre-diagnostic NSAID use, stratified analysis by pre-diagnostic use of proton pump inhibitors (PPIs)**

**Supplementary Table S4. Hazard ratios (95% confidence intervals) of depression, anxiety, and stress-related disorders during the year after cancer diagnosis in relation to pre-diagnostic use of NSAIDs, analysis by selectivity of NSAIDs**

**Supplementary Table S1.** **Hazard ratios (95% confidence intervals) of depression, anxiety, or stress-related disorder during the year after cancer diagnosis in relation to pre-diagnostic use of NSAIDs**

|  | **1000 PYs** | **Event (IR)** | **Model1** *^a^* | **Model2** *^b^* | **Model3** *^c^* |
| --- | --- | --- | --- | --- | --- |
| **Depression** | | | | | |
| No NSAIDs | 175 | 1,628 (9.3) | 1.00 | 1.00 | 1.00 |
| Aspirin | 50 | 435 (8.7) | 0.87 (0.77-0.98) | 0.88 (0.77-0.99) | 0.89 (0.79-1.01) |
| Non-aspirin NSAIDs | 44 | 524 (12.0) | 1.18 (1.07-1.31) | 1.19 (1.07-1.31) | 1.17 (1.06-1.30) |
| Both NSAIDs | 13 | 145 (11.5) | 1.05 (0.88-1.27) | 1.07 (0.89-1.28) | 1.08 (0.90-1.30) |
| **Anxiety** |  |  |  |  |  |
| No NSAIDs | 175 | 1,589 (9.1) | 1.00 | 1.00 | 1.00 |
| Aspirin | 50 | 349 (7.0) | 0.85 (0.74-0.98) | 0.82 (0.71-0.94) | 0.85 (0.74-0.98) |
| Non-aspirin NSAIDs | 44 | 551 (12.6) | 1.30 (1.18-1.43) | 1.32 (1.19-1.45) | 1.27 (1.15-1.40) |
| Both NSAIDs | 13 | 118 (9.3) | 1.04 (0.85-1.27) | 1.02 (0.83-1.25) | 1.03 (0.84-1.26) |
| **Stress-related disorders** | | | | | |
| No NSAIDs | 175 | 669 (3.8) | 1.00 | 1.00 | 1.00 |
| Aspirin | 50 | 85 (1.7) | 0.87 (0.67-1.14) | 0.86 (0.66-1.13) | 0.88 (0.67-1.16) |
| Non-aspirin NSAIDs | 44 | 218 (5.0) | 1.30 (1.11-1.51) | 1.27 (1.09-1.49) | 1.25 (1.07-1.47) |
| Both NSAIDs | 13 | 24 (1.9) | 0.85 (0.55-1.31) | 0.83 (0.54-1.28) | 0.83 (0.54-1.29) |

Abbreviations: PYs, person-years; IR, incidence rate per 1000 person-years.

Cancer patients with any diagnosis of depression, anxiety, or stress-related disorders, namely post-traumatic stress disorder, acute stress reaction, adjustment disorder or other stress reactions before cancer diagnosis were excluded from the analysis. IRs were calculated by dividing the number of patients that received a diagnosis of depression, anxiety and stress-related disorders by the number of person-years accumulated during follow-up. Separate multivariable Cox proportional hazard models were used to estimate the hazard ratios and 95% confidence intervals of depression, anxiety, or stress-related disorders in relation to exclusive NSAID use, using no use of NSAIDs as the reference.

*^a^* Estimates were adjusted for age, sex, calendar year at cancer diagnosis, educational level, occupation, region of residence, marital status, and Chronic Disease Score.

*^b^* Estimates were additionally adjusted for potential indications for NSAID use.

*^c^* Estimates were additionally adjusted for common cancer types and cancer stage, as well as subtypes of hematological malignancies (leukemia, lymphoma, myeloma, myelodysplastic syndrome, or myeloproliferative neoplasm).

**Supplementary Table S2. Hazard ratios (95% confidence intervals) of depression, anxiety, and stress-related disorders during the year after cancer diagnosis in relation to pre-diagnostic use of NSAIDs, after excluding patients with any psychiatric disorders before cancer diagnosis**

| **Groups** | **1000 PYs** | **Event (IR)** | **Model1** *^a^* | **Model2** *^b^* | **Model3** *^c^* |
| --- | --- | --- | --- | --- | --- |
| No NSAIDs | 168 | 3,065 (18.3) | 1.00 | 1.00 | 1.00 |
| Aspirin | 47 | 722 (15.2) | 0.89 (0.81-0.98) | 0.88 (0.80-0.97) | 0.90 (0.81-0.99) |
| Non-aspirin NSAIDs | 42 | 1,025 (24.6) | 1.26 (1.17-1.35) | 1.27 (1.18-1.36) | 1.23 (1.15-1.33) |
| Both NSAIDs | 12 | 227 (19.0) | 1.02 (0.88-1.18) | 1.01 (0.88-1.17) | 1.02 (0.88-1.18) |

Abbreviations: PYs, person-years; IR, incidence rate per 1000 person-years.

IRs were calculated by dividing the number of patients that received a diagnosis of depression, anxiety and stress-related disorders by the number of person-years accumulated during follow-up. Multivariable Cox proportional hazard models were used to estimate the hazard ratios and 95% confidence intervals in relation to exclusive NSAID use, using no use of NSAIDs as the reference.

*^a^* Estimates were adjusted for age, sex, calendar year of cancer diagnosis, educational level, occupation, region of residence, marital status, and Chronic Disease Score.

*^b^* Estimates were additionally adjusted for potential indications for NSAID use.

*^c^* Estimates were additionally adjusted for common cancer types and cancer stage, as well as subtypes of hematological malignancies (leukemia, lymphoma, myeloma, myelodysplastic syndrome, or myeloproliferative neoplasm).

**Supplementary Table S3. Hazard ratios (95% confidence intervals) of depression, anxiety, and stress-related disorders during the year after cancer diagnosis in relation to pre-diagnostic NSAID use, stratified analysis by pre-diagnostic use of proton pump inhibitors (PPIs)**

| **Groups** | **1000 PYs** | **Event (IR)** | **Model 1** *^a^* | **Model 2** *^b^* | **Model 3** *^c^* | **P for interaction** *^d^* |
| --- | --- | --- | --- | --- | --- | --- |
| **No PPI use** |  |  |  |  |  |  |
| No NSAIDs | 167 | 3,189 (19.1) | 1.00 | 1.00 | 1.00 | - |
| Aspirin | 46 | 705 (15.5) | 0.91 (0.82-1.00) | 0.89 (0.80-0.98) | 0.91 (0.82-1.00) | - |
| Non-aspirin NSAIDs | 40 | 989 (24.9) | 1.24 (1.15-1.33) | 1.24 (1.15-1.33) | 1.21 (1.13-1.30) | - |
| Both NSAIDs | 11 | 214 (19.3) | 1.05 (0.90-1.22) | 1.04 (0.89-1.21) | 1.05 (0.90-1.22) | - |
| **PPI use** |  |  |  |  |  |  |
| No NSAIDs | 7 | 219 (30.6) | 1.00 | 1.00 | 1.00 | - |
| Aspirin | 4 | 92 (22.5) | 0.84 (0.63-1.11) | 0.85 (0.64-1.12) | 0.87 (0.66-1.16) | 0.47 |
| Non-aspirin NSAIDs | 4 | 156 (43.1) | 1.29 (1.04-1.58) | 1.32 (1.07-1.63) | 1.30 (1.06-1.61) | 0.65 |
| Both NSAIDs | 1 | 49 (32.7) | 1.10 (0.78-1.53) | 1.13 (0.81-1.59) | 1.12 (0.80-1.57) | 0.97 |

Abbreviations: PPI, Proton pump inhibitors; PYs, person-years; IR, incidence rate per 1000 person-years.

PPI use were defined as at least two dispensations of PPIs during the year before cancer diagnosis. IRs were calculated through dividing the number of patients receiving a diagnosis of depression, anxiety and stress-related disorders by number of person-years accumulated during follow-up. Separate multivariable Cox proportional hazard models were fitted for those who used and the ones that did not use PPIs, to estimate the hazard ratios and 95% confidence intervals in relation to exclusive NSAID use, using no use of NSAIDs as the reference.

*^a^* Estimates were adjusted for age, sex, calendar year of cancer diagnosis, educational level, occupation, region of residence, marital status, and Chronic Disease Score.

*^b^* Estimates were additionally adjusted for potential indications for NSAID use.

*^c^* Estimates were additionally adjusted for common cancer types and cancer stage, as well as subtypes of hematological malignancies (leukemia, lymphoma, myeloma, myelodysplastic syndrome, or myeloproliferative neoplasm).

*^d^* To assess the interaction between NSAID use and PPI use, we included an interaction term between PPI use and NSAID use in multivariable model 3 and tested for the null hypothesis that interaction parameter is equal to zero. P < 0.05 indicates a statistically significant interaction.

**Supplementary Table S4. Hazard ratios (95% confidence intervals) of depression, anxiety, and stress-related disorders during the year after cancer diagnosis in relation to pre-diagnostic use of NSAIDs, analysis by selectivity of NSAIDs**

| **Groups** | **1000 PYs** | **Event (IR)** | **Model1** *^a^* | **Model2** *^b^* | **Model3** *^c^* |
| --- | --- | --- | --- | --- | --- |
| No NSAIDs | 174 | 3,408 (19.6) | 1.00 | 1.00 | 1.00 |
| COX-1 selective | 52 | 852 (16.5) | 0.92 (0.84-1.00) | 0.91 (0.83-0.99) | 0.93 (0.85-1.01) |
| COX-2 selective | 21 | 552 (26.5) | 1.29 (1.18-1.41) | 1.30 (1.18-1.42) | 1.26 (1.15-1.38) |
| Nonselective/both NSAIDs | 33 | 801 (24.0) | 1.16 (1.07-1.26) | 1.17 (1.08-1.27) | 1.15 (1.06-1.25) |

Abbreviations: PYs, person-years; IR, incidence rate per 1000 person-years; COX, cyclooxygenase.

COX-1 selective (aspirin, flurbiprofen, ketoprofen, fenoprofen, tolmetin, oxaprozin), and COX-2 selective (coxibs, meloxicam, etodolac, mefenamic acid, diclofenac) NSAIDs were identified using [Anatomical Therapeutic Chemical Classification System](https://en.wikipedia.org/wiki/Anatomical_Therapeutic_Chemical_Classification_System), ATC codes. Nonselective NSAIDs and use of both categories were collapsed into an additional group. IRs were calculated through dividing the number of patients receiving a diagnosis of depression, anxiety and stress-related disorders by number of person-years accumulated during follow-up. Multivariable Cox proportional hazard models were used to estimate the hazard ratios and 95% confidence intervals in relation to exclusive NSAID use by selectivity of NSAIDs, using no use of NSAIDs as the reference.

*^a^* Estimates were adjusted for age, sex, calendar year at cancer diagnosis, educational level, occupation, region of residence, marital status, and Chronic Disease Score.

*^b^* Estimates were additionally adjusted for potential indications for NSAID use.

*^c^* Estimates were additionally adjusted for common cancer types and cancer stage, as well as subtypes of hematological malignancies (leukemia, lymphoma, myeloma, myelodysplastic syndrome, or myeloproliferative neoplasm).
